# Self-Management Education Through mHealth: Review of Strategies and Structures

**DOI:** 10.2196/10771

**Published:** 2018-10-19

**Authors:** Nazli Bashi, Farhad Fatehi, Mina Fallah, Darren Walters, Mohanraj Karunanithi

**Affiliations:** 1 Australian eHealth Research Centre Commonwealth Scientific and Industrial Research Organisation (CSIRO) Herston Australia; 2 Faculty of Medicine The University of Queensland Brisbane Australia; 3 Center for Online Health The University of Queensland Brisbane Australia; 4 Tehran University of Medical Sciences Tehran Islamic Republic Of Iran; 5 Department of Cardiology Prince Charles Hospital Brisbane Australia

**Keywords:** health education, mHealth, mobile apps, mobile phone, patient education, self-management education

## Abstract

**Background:**

Despite the plethora of evidence on mHealth interventions for patient education, there is a lack of information regarding their structures and delivery strategies.

**Objective:**

This review aimed to investigate the structures and strategies of patient education programs delivered through smartphone apps for people with diverse conditions and illnesses. We also examined the aim of educational interventions in terms of health promotion, disease prevention, and illness management.

**Methods:**

We searched PubMed, Cumulative Index to Nursing and Allied Health Literature, Embase, and PsycINFO for peer-reviewed papers that reported patient educational interventions using mobile apps and published from 2006 to 2016. We explored various determinants of educational interventions, including the content, mode of delivery, interactivity with health care providers, theoretical basis, duration, and follow-up. The reporting quality of studies was evaluated according to the mHealth evidence and reporting assessment criteria.

**Results:**

In this study, 15 papers met the inclusion criteria and were reviewed. The studies mainly focused on the use of mHealth educational interventions for chronic disease management, and the main format for delivering interventions was text. Of the 15 studies, 6 were randomized controlled trials (RCTs), which have shown statistically significant effects on patients’ health outcomes, including patients’ engagement level, hemoglobin A_1c_, weight loss, and depression. Although the results of RCTs were mostly positive, we were unable to identify any specific effective structure and strategy for mHealth educational interventions owing to the poor reporting quality and heterogeneity of the interventions.

**Conclusions:**

Evidence on mHealth interventions for patient education published in peer-reviewed journals demonstrates that current reporting on essential mHealth criteria is insufficient for assessing, understanding, and replicating mHealth interventions. There is a lack of theory or conceptual framework for the development of mHealth interventions for patient education. Therefore, further research is required to determine the optimal structure, strategies, and delivery methods of mHealth educational interventions.

## Introduction

### Health Education

Health education is a key strategy in the process of acquisition of behaviors that promote and maintain health; it has serious implications for health promotion, disease prevention, and illness management. According to the World Health Organization, health promotion is defined as the process of enabling the general public to improve their own health and covers a broad range of social and environmental interventions. These interventions are developed to improve individuals’ health and quality of life by addressing and preventing the underlying causes of illnesses and not merely focusing on treatment and cure. Health promotion consists of educational strategies to inform people of what they can do to stay healthy and to address the issues in the community that influence mostly health and well-being [1].

Disease prevention is an individual or group-based intervention for primary and secondary prevention, which aims to decrease the burden of diseases and associated risk factors. While primary prevention is defined as actions taken to avoid the manifestation of a disease, secondary prevention consists of interventions for early detection, which may improve patients’ health outcomes [1]. Education on secondary prevention and illness management for patients with chronic disease who require day-to-day self-monitoring and symptom recognition is vital. Furthermore, it is important for patients to not only attain knowledge but also involve in the process of care and gain empowerment over their conditions. As a result, the provision of information, knowledge, self-management skills, and self-efficacy encouragement to patients is essential to produce active participation and consequently improve their health outcomes [2-4]. Health care providers play an integral role in collaborating with their patients to enhance knowledge, develop skills, and build confidence.

For those with chronic diseases, patient education is focused on alleviating complications and optimizing the quality of life. Hence, patient education is directed toward behavioral change, and the development of essential skills and knowledge for self-management [5,6]. The focus of behavioral change is either the adoption of new behavior, such as daily exercise or the discontinuation of old behavior, such as smoking. Weight loss, smoking cessation, and increasing physical activity levels are the main target areas for behavior change of chronic patients [6].

To manage chronic conditions, in addition to education, patients require long-term support to improve their self-management skills and achieve the desired behavioral change [7,8]. Self-management education (SME) has been recognized as a complementary intervention for fostering behavioral changes; this has been supported by the internationally influential Chronic Care Model [7]. It is now evident that the efficient provision of SME is challenging for many health care professionals. Clinicians pointed out the lack of time, competing demands, health care systems that are structured to focus on individual conditions rather than multiple comorbidities, and limited patient motivation as barriers for the implementation of SME [9,10]. Previous research identified effective strategies for educating patients and providing SME [6,7]. The implementation of such interventions and tailoring them based on patients’ needs and preferences demand significant resources and multiple face-to-face educational sessions, which traditionally happen in health care settings [11].

### mHealth For Patient Education

Information and communications technologies and digital devices, such as smartphones, offer a potentially powerful means for patient education and behavioral change reinforcements [12-14]. A large number of health-related software apps have been designed and are available for both health care professionals and patients [15]. The number of medical or health-related apps in the major app stores (Apple App Store and Google Play Store) is increasing rapidly [16]. The use of smartphones is changing the provision of patient education in health care through convenient, individually tailored, and contextually meaningful delivery of interventions [17]. Furthermore, smartphone apps have lower costs, reduce the burden on patients, and overcome some limitations of traditional in-person interventions [18].

A number of systematic reviews and meta-analyses have investigated the effect of smartphone apps on patients and consumers health outcomes [19-21]. In addition, a broad range of smartphone educational apps has been used to improve public health knowledge. However, these interventions are not found equally effective [22]. Effective patient education strategies were identified as traditional lectures, discussions, simulated games, computer technology, written material, audiovisual sources, verbal recall, demonstration, and role-playing [23]. Research showed that internet-based interventions, which were developed based on a theory, were more effective than those with no theoretical basis [24]. None of the existing literature reviews have investigated mHealth SME interventions with respect to the evidence around the theoretical frameworks. Furthermore, a limited number of studies on smartphone apps provided sufficient information related to educational contents and methods of delivering such interventions. Hence, this review aims to investigate smartphone-based educational interventions for patient self-management. The review also aims to explore the structures and strategies (including format, interactivity, use of theory, duration of education, and health care professionals’ follow-up) of the educational interventions along with any documented theory or framework, which informed the design of such interventions. To investigate the studies, we categorized the aim of patient education with respect to World Health Organization health education interventions into health promotion, disease prevention, and illness management.

## Methods

### Search Strategy

We conducted a comprehensive electronic search of 4 major biomedical databases (PubMed, Cumulative Index to Nursing and Allied Health Literature, Embase, and PsycINFO) for peer-reviewed papers published from 2006 to 2016. A sensitive search strategy was developed by a combination of controlled vocabulary (Medical Subject Headings terms) and free text terms according to recent recommendations for searching the PubMed database [25-27]. The electronic search incorporated 3 main concepts: (1) mHealth; (2) patient education; and (3) self-management (see Multimedia Appendix 1). The search strategy was modified specifically for every other database based on their individual guide. Furthermore, search results were downloaded to EndNote citation manager software, and the duplicates were removed.

### Inclusion and Exclusion Criteria

The criteria for considering studies for this review were as follows.

#### Design

We considered peer-reviewed studies for inclusion. Primary or secondary studies reporting clinical trials were included regardless of their study design, except for case reports. We reviewed papers with a broad range of methodology, including qualitative and quantitative. However, conference abstracts, book reviews, letters, editorials, and unpublished studies were excluded.

#### Participants

We considered patients with diverse conditions regardless of their age, gender, or ethnicity in this review. However, the authors might have established the diagnostic criteria in their respective papers.

#### mHealth Educational Interventions

Any mHealth educational intervention designed or delivered for health promotion, disease prevention, or illness management was included. We considered interventions that consisted of educational modules or materials either as the main intervention or part of health care delivery for patients with chronic conditions.

### Data Extraction and Synthesis

Three reviewers extracted data from the final set of included papers. In case of discrepancy, the reviewers discussed the issues and reached an agreement. Owing to resource limitation, we excluded papers published in other than the English language. Data extracted from each paper were summarized in 3 separate tables. Multimedia Appendix 2 reports study characteristics and includes the name of the first author, study design, disease or condition, aims of education, sample size, theory-based and description of the app. Study designs were categorized as randomized controlled trials (RCTs), case-control, proof-of-concept, or pilot research. In addition, we categorized the aims of education to health promotion, disease prevention, and illness management. Table 1 details the results of the RCTs. Multimedia Appendix 3 was used to detail intervention strategies and structures, including educational topics, modes of delivery, and measurement tools.

### Literature Search Results

**Figure 1:**
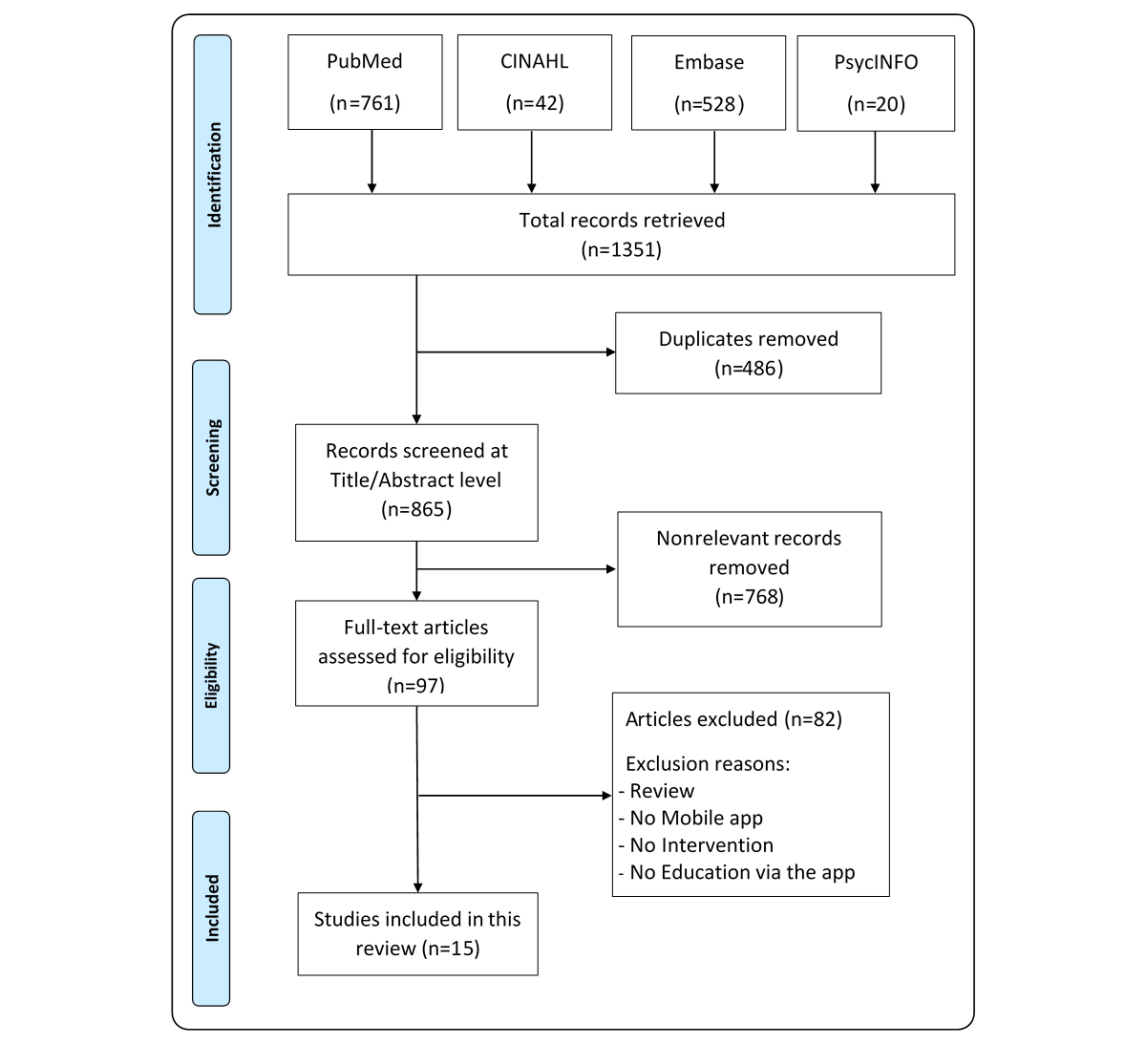
Study flow diagram. CINAHL: Cumulative Index to Nursing and Allied Health Literature.

The Preferred Reporting Items for Systematic Reviews and Meta-Analyses flow diagram was used to document the paper selection process (Figure 1). A total of 1351 papers were retrieved from the electronic search of 4 databases. After removing duplicate records, we screened 865 records at the title or abstract level and examined the full text of 97 potentially relevant papers. Finally, we included 15 studies in this review.

**Table 1:** Summary of the intervention and results of the included randomized controlled trials.

Study	Study design	Disease or condition	Primary outcome	Follow-up	Results
Ledford et al, 2016 [28]	Pilot RCT^a^	Pregnancy	Patient activation	32 weeks	There was a statistically significant difference in the patient activation between notebook and mobile groups (*P*=.02).
Zhou et al, 2016 [29]	Open RCT	Diabetes	Hemoglobin A_1c_	3 months	Diabetic patients in the intervention group (using the Welltang app) achieved statistically significant improvements in hemoglobin A_1c_ (*P*<.001).
Direito et al, 2015 [30]	RCT (3-arm)	Physical activity	Cardiorespiratory fitness	8 weeks	There was no significant intervention effect on the primary outcome using either of the apps.
Fukuoka et al, 2015 [31]	RCT	Overweight	Percentage change in weight and body mass index	5 months	There was a statistically significant difference in weight loss between the intervention and control groups (*P*<.001).
Depp et al, 2015 [32]	RCT	Bipolar disorder	Depressive symptoms	24 weeks	Participants in the intervention group showed significantly greater reductions in depressive symptoms after 6 and 12 weeks (Cohen *d* values for both were.48). However, these effects were not maintained at 24-week follow-up.
Ly et al, 2014 [33]	Open RCT	Depression	Depression	6 months	No significant interaction effects of group and time on the Patient Health Questionnaire-9 and the Beck’s Depression Inventory-II were found between the groups, either from pretreatment to posttreatment.

^a^RCT: randomized controlled trial.

### Quality Assessment

We graded the quality of the evidence using the new mHealth evidence and reporting assessment (mERA) checklist [34]; this checklist has been developed by the World Health Organization mHealth Technical Evidence Review Group. mERA guides in assessing the reporting quality of mHealth interventions covering both feasibility and effectiveness studies. The checklist is a valuable tool and consists of 16 essential criteria to support the completeness of reporting and replication of the mHealth interventions by addressing its content, context, and implementation features. Effective and comprehensive reporting may help to improve the program design, foster collaboration among service providers, reduce duplication of efforts, and ultimately increase the impact and ability to scale effective mHealth interventions. Furthermore, the checklist has been previously used to assess the reporting quality of mHealth interventions [35].

## Results

### Study Characteristics

Multimedia Appendix 2 summarizes the study characteristics of mHealth educational interventions. Overall, 115 papers have reported on the use of smartphone apps for patient education. Of these, 3 studies targeted cardiovascular disease, including cardiac rehabilitation, coronary artery disease, and heart failure [36-38]; 3 studies focused on overweight adults and physical activity [30,31,39]; 2 smartphone apps reported on asthma [40,41]; and 3 studies targeted mental illnesses such as depression and bipolar disorder [32,33,42]. Diabetes was the focus of 2 mobile-based interventions [29,43]. Furthermore, 1 study reported the feasibility of engaging adolescents with smartphone education [44], and 1 study focused on parental education and engagement [28]. Papers included in the review reflected study designs across the research spectrum including RCTs (n=6) [28-33] and case-control (n=1) [39], proof-of-concept (n=2) [36,40], and feasibility and pilot studies (n=5) [37,41-44]. One paper did not report the design of the study [38]. Sample sizes were diverse and ranged from 10 participants who were engaged in heart failure education [36] to 173 pregnant women for parental education [28].

As shown in Table 1, a number of primary outcomes were measured in 6 RCTs, including clinical health, patient activation, or psychological indicators [28-31,33]. In 1 study, a group of pregnant women had a statistically significant (*P*=02) higher level of patient engagement than their comparators [28]. In another study on diabetes, the intervention group that used a smartphone app achieved lower glucose levels than the control group (*P*<.001) [29]. A 3-arm RCT comparing 2 smartphone apps reported no significant intervention effect on the cardiorespiratory fitness level of young adults [30]. An RCT of a novel smartphone app recruited overweight adults who were at risk of diabetes, and the results showed that participants in the intervention group lost an average of 6.2 kg (SD 5.9) between the baseline and 5-month follow-up compared with the control group’s gain of 0.3 kg (SD 2.7; *P*=.001) [31]. In addition, 2 studies measured patients’ depressive symptoms as primary outcomes. While 1 study reported a statistically significant (Cohen *d*=.48) reduction of depressive symptoms in the intervention group [32], the other reported no significant effect of intervention [33].

### Quality of Evidence

**Figure 2:**
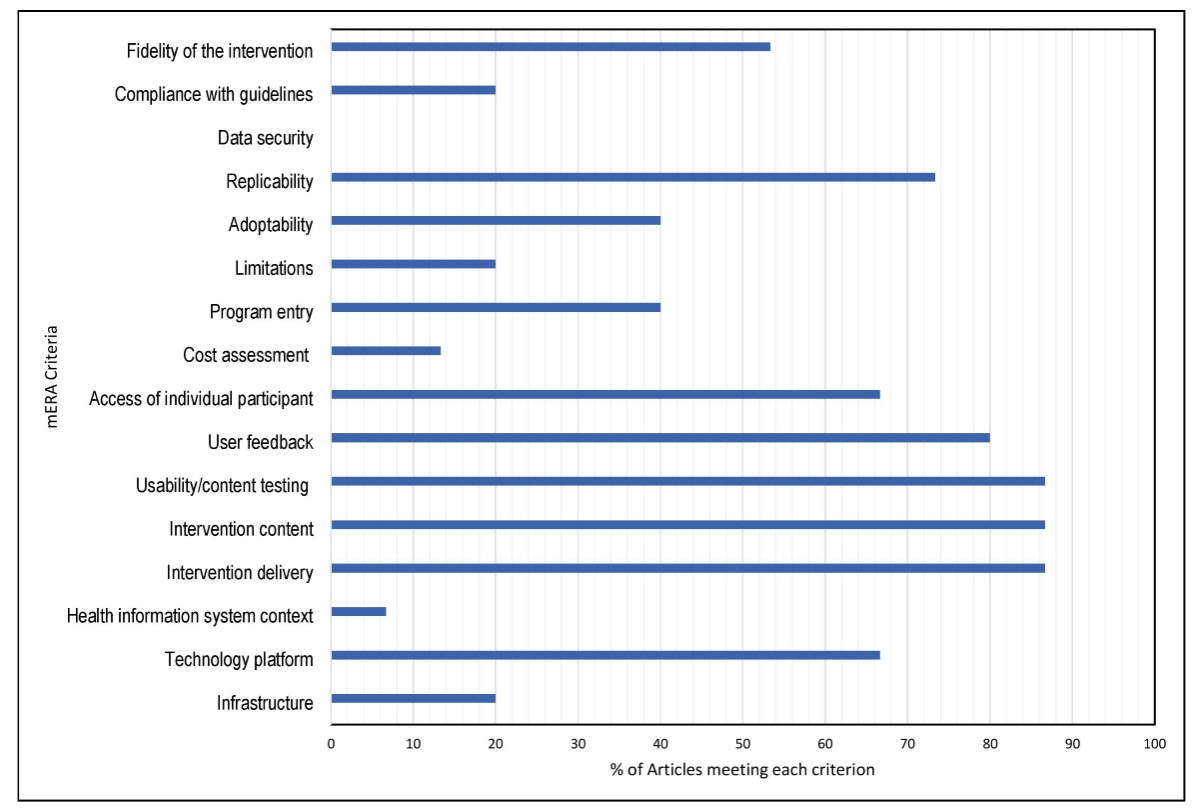
The mHealth evidence and reporting assessment checklist. mERA: mHealth evidence and reporting assessment.

Based on the mERA criteria for the quality of reporting in mHealth, (13/15, 86%) of included studies reported on the content of smartphones interventions, modes of delivery, and testing usability. Of 15, none of the studies reported on the measures taken to protect data security, privacy, and confidentiality. While (12/15, 80%) of studies reported on users’ feedback, (6/15, 40%) described patient or user satisfaction. Only (2/15, 13%) of the studies provided some level of information on the cost associated with the development or delivery of mHealth interventions (Figure 2).

### Educational Aims

Smartphone interventions identified in this review reflected on 3 different aspects of health education, including health promotion, disease prevention, and illness management.

#### Health Promotion

A total of 3 smartphone interventions targeted health promotion in adolescent and pregnant women. It is evident that pregnant women who used a mobile app for prenatal education and engagement were more engaged than their control counterparts (pregnant women who used a notebook) [28]. Kenny et al [44] reported that the overall engagement was high in the health promotion program for adolescents.

#### Disease Prevention

Of the 15 studies, 2 aimed to reduce diseases’ risk factors (ie, primary prevention) using smartphone apps; these studies focused on overweight participants and showed a reduction in weight and blood glucose levels [31,39].

#### Illness Management

Heart failure, coronary heart disease, diabetes, and asthma were the main chronic diseases reported in the included papers [36-38,40,41,43]. In addition, we considered mobile-based education for mental illnesses such as bipolar disorder and depression as part of illness management [32,33,42]. Reviewed studies that focused on illness management (ie, secondary prevention) reported interventions on different aspects of education such as knowledge, self-management skills, and behavioral change interventions.

### Structure of Interventions and Delivery Strategies

A total of 8 studies reported that their interventions were developed by the research team and health care professionals [31,33,36-38,40-42]. The educational materials covered by the smartphone apps were diverse and consisted of broad topics such as causes of diseases, monitoring signs and symptoms, exercise instructions, diet recommendations, and coping strategies. Random coping tips were provided to adolescents and covered “think positively” themes and emotional self-monitoring advice [44]. The most commonly used format of educational materials was text [28,32,33,36-40,44]. In addition, video clips [31,37,40,41,43] and audio files [30,36] were used in 7 studies. With regard to the knowledge assessment, 2 studies reported the assessment of patients’ knowledge following the educational intervention [29,36]. In 1 study [36], authors developed a feature in the smartphone apps for users to assess their knowledge, and in another study authors used the diabetes knowledge survey [29] (Multimedia Appendix 3).

### Theoretical Frameworks for the Development of the App

In total, 4 studies reported using a theory or a conceptual model to underpin the educational materials of smartphones apps [33,36,38,41]. Athilingam et al [36] used a number of theories, including Mayers’ cognitive theory of multimedia learning, Swellers’ cognitive load theory, industrial design approach utilizing a pedagogical agent and problem-based learning, for designing smartphone apps for patients with heart failure. Behavioral activation was used to develop apps for patients with depression [33]. Technology acceptance model and analysis, design, development, implementation, evaluation model were used in 2 studies [38,41].

## Discussion

### Principal Findings

This study reviewed 15 studies on innovative educational interventions using smartphone apps for participants with diverse conditions. Interventions that were identified in this review aimed to deliver educational materials through smartphones to promote health, prevent diseases, and manage chronic illnesses. The results of our review showed that mHealth interventions were mainly focused on the illness management of patients with chronic disease. Although we considered self-management as one of the key constructs of our search strategy, none of the studies included in this review formally assessed self-management as the primary outcome. However, behavioral change, as an indirect outcome of self-management, was assessed by a number of studies.

Although short message service were the most common format used to deliver educational materials through smartphones, 7 studies used audio or visual aids. The use of audio or visual format provides an additional means of communication for conveying educational information that may be difficult to communicate through words alone. Furthermore, audio or visual educational aids may increase patient understanding of a particular situation or specific procedure [2]. Yet, there remains limited knowledge on the best format of smartphone communication for patient education. As smartphone adoption is rapidly increasing, health care professionals should give more consideration to the development and evaluation of audio or visual materials for patient education.

The findings from the reviewed studies highlighted the fact that there is insufficient evidence to inform the underpinning theory or framework in the development of current smartphones apps. In many studies, the theoretical rationale for the development of apps and various components of the intervention, including educational materials, were not reported (Multimedia Appendix 2). The application of the theory is widely recognized as a crucial component of health interventions, and it is evidenced that strong theory is critical in identifying the effectiveness of specific components of interventions and optimizing their intensity [45]. However, the role of theory in developing mobile-based interventions with educational components has been largely disregarded.

Surprisingly, only 2 papers included in this review utilized measurement tools to assess participants’ knowledge following their interventions. As patients can control their illness and limit worsening symptoms when they understand the principles of chronic disease management and learn to undertake simple interventions [46], the evaluation of educational interventions must be an integral part of practitioners plan for education. The assessment of knowledge completes a feedback loop, enabling health care professionals to determine the intervention’s effectiveness. If the intervention did not have the intended effect, the content or delivery methods of the educational intervention may need to be modified to improve their effectiveness [47]. A previous systematic review identified patients’ recall as an effective teaching strategy [23]. Hence, we recommend assessing patients’ knowledge following an educational intervention.

As shown in Figure 2, an average of (42/100, 42.0%) of recommended essential criteria for reporting mHealth were met. Although most ethical organizations are now requiring researchers to provide reports on the details of steps taken for maintaining data security and confidentiality, no study has reported about it. Notably, only (3/15, 20%) of studies reported on the compliance of their interventions with national guidelines. It can be concluded that there is a lack of evidence to support the use of national guidelines or other authoritative sources of information for the development of mobile-based interventions for patient education. Nevertheless, the mERA checklist is relatively new; therefore, the low percentage of met criteria for reporting in several mHealth studies should not be surprising [34].

Of the 15 studies included in this review, 6 were RCTs that examined a range of health outcomes on patients with different conditions. The results of 4 RCTs showed statistically significant effects of smartphone-based interventions on health outcomes, including the patient engagement level, hemoglobin A_1c_, weight loss, and depression. However, the 3-arm RCT evaluating the smartphone intervention for improving young adults’ physical activity did not show statistically significant effect on cardiorespiratory fitness and physical activity level. Furthermore, a smartphone app based on the behavioral activation did not show a statistically significant reduction on major depression disorder.

Although the results of RCTs were mainly positive, studies varied significantly with regard to mHealth educational interventions. Furthermore, only one RCT reported the theoretical underpinning of the educational intervention, and this highlights the lack of theory in developing and evaluating mHealth interventions. As the reporting quality of reviewed studies was poor, it was impossible to compare the effects of interventions based on their educational interventions’ structures and strategies.

### Conclusions

The results of this review generally support that patients with diverse conditions benefit from mobile-based educational interventions. However, we were unable to identify any effective specific structure or strategy for the delivery of such interventions owing to the scarcity of high-quality studies and suboptimal reporting quality of the reviewed papers. Thus, additional research is needed to determine the optimal structure, format, and delivery methods for educational instructions that are used in mHealth interventions for patient education. We strongly recommend adoption of standard tools, such as mERA essential criteria, for reporting mHealth interventions. This will facilitate better reporting and improve the ability to synthesize the evidence in future.

## Appendix

Multimedia Appendix 1 Medical Subject Headings (MeSH) terms and free-text keywords used for the PubMed search.

Multimedia Appendix 2 Characteristics of the included studies describing mobile apps for self-management education of chronic patients.

Multimedia Appendix 3 Description of the interventions strategies for self-management.

## Abbreviations

mERA: mHealth evidence and reporting assessment
RCT: randomized controlled trial
SME: self-management education
