# Supplementary material for: Self-Management Education Through mHealth: Review of Strategies and Structures
Source: JMIR Mhealth Uhealth. 2018 Oct 19;6(10):e10771. doi: 10.2196/10771 (PMC6239867; doi:10.2196/10771)
Supplement: Multimedia Appendix 1 [file mhealth_v6i10e10771_app1.pdf]

Appendix. Search strategy for PubMed

Concept	Search query
Patient education	"Patient Education as Topic"[Mesh]<br>"Counseling"[Mesh]<br>"Education, Distance"[Mesh]<br>"Health Education"[Mesh]<br><br>Patient education<br>Patient counselling<br>Patient counseling<br>Patient training<br>Patient teaching<br>Health education<br>Health promotion
Mobile health	Mobile Applications[Mesh]<br>Smartphone[Mesh]<br>Cellular Phone[Mesh]<br>Computers, Handheld[Mesh]<br>NOT "Text Messaging"[Majr]<br><br>Cellphone/Cellphones<br>Cell phone/Cell phones<br>Cellular phone/Cellular phones<br>Mobile phone/Mobile phones<br>Mobile device/Mobile devices<br>Mobile-based<br>Mobile telephone/Mobile telephones<br>Smartphone/Smartphones<br>Smart phone/Smart phones<br>iPhone<br>Android<br>Mhealth/m-health<br>mobile health
Self-management	"Self Care"[Mesh]<br><br>Self-care<br>Self-management<br>Self-efficacy<br>Patient centred care<br>Behaviour therapy<br>Exercise therapy<br>Coping strategies
