# Supplementary material for: Self-Management Education Through mHealth: Review of Strategies and Structures
Source: JMIR Mhealth Uhealth. 2018 Oct 19;6(10):e10771. doi: 10.2196/10771 (PMC6239867; doi:10.2196/10771)
Supplement: Multimedia Appendix 2 [file mhealth_v6i10e10771_app2.pdf]

Multimedia Appendix 2. Characteristics of the included studies describing mobile apps for self-management education of chronic patients.

Study	Study design	Sample size	Disease/Condition	Aims of patient education	Description of the app	Theory or model that was used as the basis of the intervention
Athilingam 2016	Proof-of-concept	10	Heart failure	Illness management	Information, scenario, and avatar were used to develop a mobile platform	Mayers' Cognitive theory of multimedia learning, Swellers' cognitive load theory, industrial design approach utilizing a pedagogical agent and problem-based learning
Cook 2016	Proof-of-concept	60	Asthma	Illness management	Self-assessment asthma survey, personalized coaching, 48 unique videos, alerts	Not reported
Hidalgo-Mazzei 2016	Feasibility study	52	Bipolar Disorders	Illness management	NR[a]	NR
Ly 2014	RCT[b]	81	Depression	Illness management	Smartphone app used for step-by-step behavioral intervention and education	Behavioral activation
Ledford 2016	Pilot RCT	173	Pregnancy	Health promotion	Smartphone app was used for education and record-keeping	NR
Zhou 2016	RCT	100	Diabetes	Illness management	Smartphone app used for diabetes management	NR
Bain 2015	Feasibility	47	Diabetes	Illness management	Smartphone was used to deliver one-way video transmission	NR
Direito 2015	RCT (3-arm)	51	Physical Activity	Health promotion	Commercially available apps were used including Zombies, Run, and Get Running	NR
Fukuoka 2015	RCT	61	Overweight	Disease prevention	Diabetes intervention combined with a mobile app was used	NR

Study	Study design	Sample size	Disease/Condition	Aims of patient education	Description of the app	Theory or model that was used as the basis of the intervention
Kenny 2015	Feasibility	43	Adolescent	Health promotion	Mobile app used to improve mental health	NR
Cho 2014	NR	30	Coronary artery disease	Illness management	An educational smartphone app was used as a postdischarge tool for patients with coronary artery disease	Analysis, Design, Development, Implementation, Evaluation model
Depp 2015	RCT	82	Bipolar disorder	Illness management	Mobile phone intervention was used to educate self-management of mood changes and symptoms	NR
Forman 2014	Pilot study	26	Cardiac rehabilitation	Illness management	Participant completed a daily “task list” including medication, walking, education and survey	NR
Haze 2013	Pilot study	25	Asthma	Illness management	Smartphone app was used to educate teenagers with asthma	Technology Acceptance Model
Lee 2010	Case–control	36	Overweight	Disease prevention	Interactive mobile phone based app was used to analyze the daily nutrition intake and patterns of daily exercise	NR

[a]NR: not reported.

[b]RCT: randomized controlled trial.
