# Supplementary material for: Self-Management Education Through mHealth: Review of Strategies and Structures
Source: JMIR Mhealth Uhealth. 2018 Oct 19;6(10):e10771. doi: 10.2196/10771 (PMC6239867; doi:10.2196/10771)
Supplement: Multimedia Appendix 3 [file mhealth_v6i10e10771_app3.pdf]

Multimedia Appendix 3. Description of the interventions strategies for self-management education.

Study	Duration; frequency of intervention	Educational topics	Contents prepared by Health care provider; Researcher	Format of contents; Time	Interactivity with Heath care provider; Follow-up	Study results
Athilingam 2016	2 hours of beta testing the app; One time off test by patients	HF, Causes and types, Care, Symptoms, Feeling, low-salt diet, exercise, medication, heart-brain connection, other chronic conditions	Patients and experts (physicians, nurse practitioners and nurses)	Text, audio; NR[a]	Avatar; NR	Patients were confident in using the app; 95% of patients reported being very likely to use the app.
Cook 2016	4 months; on demand	Triggers, medication, management of acute symptoms	Clinicians	Text, video; NR	NR; Yes	Improved asthma control and patients reported high satisfaction.
Hidalgo-Mazzei 2016	3 months; daily	Psychoeducational	Bipolar disorder experts	Text (<100 words each); adjustable	Basic (if needed); Yes	The experience using the application was satisfactory.
Ly 2014	8 weeks; daily	In addition to the Web-based education, smartphone app was used to educate 54 behaviors	Study research group	Text; NR	BA app: 20 min per week per participant; MA app: no communication; Yes, with messaging	Significant effects on primary outcomes were reported.
Ledford 2016	32 weeks; NR	NR	NR	Text; NR	NR; Yes	Mobile app users were more engaged and activated (*P*<.2). The intervention group had higher frequency usage of educational tools (*P*<.4)
Zhou 2016	3 months; NR	Diabetic knowledge on diet, exercise, medicine, and blood glucose monitoring	NR	NR; NR	High; Yes	Mobile app users had better glycemic control, improved knowledge and self-care behavior.
Bain 2015	8 weeks; alternate week	NR	NR	Video; NR	High; Yes	User satisfaction was assessed. On average, 82% of participants responded positively on their satisfaction with Tele-DSME.
Direito 2015	8 weeks; 3 times per week	Running techniques, instructions on how to perform the training components	NR	Text, Audio; NR	NR; Up to 3 visits (overall)	Apps did not have significant effect on physical activity.
Fukuoka 2015	5 months; daily	Complement to in-person training	Research team	Text messages and video clips; 11 am	NR; Yes	Mean 6.8% weight loss over 5-month intervention
Kenny 2015	One week; Tips appeared when participant rated his or her mood	Coping tips based on "think positively" course and emotional self-monitoring based on coping strategies	NR	Text; NR	Low; NR	Overall participants' engagement with the app was measured.

Study	Duration; frequency of intervention	Educational topics	Contents prepared by Health care provider; Researcher	Format of contents; Time	Interactivity with Heath care provider; Follow-up	Study results
Cho 2014	NR; once	CAD[b], daily life management, risk factors, drug management, dietary management, and exercise management	Nurse researchers	Text; NR	NR; NR	High level of satisfaction among patients with CAD
Depp 2015	Four face-to-face sessions, then assigned to paper and pen mood monitoring or mobile delivered self-management strategies; daily	Coping strategy provided. This was complementary to the face-to-face educational sessions.	NR	Text; Morning and evening	Low; NR	Web-based Education was conducted through a smartphone.
Forman 2014	30 days; daily	NR	The Wellframe team developed the content, and cardiac rehabilitation providers endorsed the program.	Text, videos; NR	High (personalized feedback); NR	The intervention improved the patients' compliance and adherence.<br>Providers reported that intervention enhanced their provision of therapy by improving communication, clinical insight, patient participation, and program efficiency.
Haze 2013	NR; daily	Short education modules, personalized asthma action plan, questionnaire to assess asthma control skills	Health care providers	Video, slide; NR	Medium; Yes	Smartphone technology and text messaging improved the nurse-patient relationship.
Lee 2010	6 weeks; NR	Diet planner; Diet game provided a quiz based learning tool, knowledge about nutrition	NR	Text (quiz); NR	Low; No	Participants receiving the Smart Care service had lower blood glucose and HbA1c during 6 months follow-up (*P*<.001)

[a]NR: Not Reported; [b]CAD: Coronary Artery Disease
